# Cost-effectiveness of psilocybin-assisted therapy for severe depression: exploratory findings from a decision analytic model

**DOI:** 10.1017/S0033291723001411

**Published:** 2023-12

**Authors:** Paul McCrone, Henry Fisher, Clare Knight, Rebecca Harding, Anne K. Schlag, David J. Nutt, Joanna C. Neill

**Affiliations:** 1 Institute for Lifecourse Development, University of Greenwich, London, UK; 2 Clerkenwell Health, London, UK; 3 Clinical Psychopharmacology Unit, University College London, London, UK; 4 Drug Science, London, UK; 5 Psychedelic Research Group, Centre for Neuropsychopharmacology, Division of Brain Sciences, Faculty of Medicine, Imperial College London, London, UK; 6 Division of Pharmacy and Optometry, School of Health Sciences, The University of Manchester, Manchester, UK

**Keywords:** Decision analysis, psilocybin, psychedelic assisted psychotherapy (PAP), treatment-resistant depression (TRD)

## Abstract

**Background:**

There is growing evidence to support the use of the psychedelic drug psilocybin for difficult-to-treat depression. This paper compares the cost-effectiveness of psilocybin-assisted psychotherapy (PAP) with conventional medication, cognitive behavioural therapy (CBT), and the combination of conventional medication and CBT.

**Methods:**

A decision model simulated patient events (response, remission, and relapse) following treatment. Data on probabilities, costs and quality-adjusted life years (QALYs) were derived from previous studies or from best estimates. Expected healthcare and societal costs and QALYs over a 6-month time period were calculated. Sensitivity analyses were used to address uncertainty in parameter estimates.

**Results:**

The expected healthcare cost of PAP varied from £6132 to £7652 depending on the price of psilocybin. This compares to £3528 for conventional medication alone, £4250 for CBT alone, and £4197 for their combination. QALYs were highest for psilocybin (0.310), followed by CBT alone (0.283), conventional medication alone (0.278), and their combination (0.287). Psilocybin was shown to be cost-effective compared to the other therapies when the cost of therapist support was reduced by 50% and the psilocybin price was reduced from its initial value to £400 to £800 per person. From a societal perspective, psilocybin had improved cost-effectiveness compared to a healthcare perspective.

**Conclusions:**

Psilocybin has the potential to be a cost-effective therapy for severe depression. This depends on the level of psychological support that is given to patients receiving psilocybin and the price of the drug itself. Further data on long-term outcomes are required to improve the evidence base.

## Introduction

Major depressive disorder (MDD) is common and results in substantial disease and economic burden (Arias-de la Torre et al., 2021). While around one-third of people who experience MDD will recover after one episode, for others the course will be worse and many will not fully recover (Eaton et al., 2008). The costs of depression are substantial to the individual, their families, as well as society, and are elevated especially in cases of treatment-resistant depression (TRD) (McCrone, Dhanasiri, Patel, Knapp, & Lawton-Smith, 2008; McCrone et al., 2018). While there are established treatment options for many with MDD (National Collaborating Centre for Mental Health, 2010), the effectiveness of therapeutic options for TRD is more limited.

There has been a recent resurgence of interest in the potential use of psychedelic substances to treat a variety of psychiatric and neurological disorders (Carhart-Harris & Goodwin, 2017; Goldberg, Pace, Nicholas, Raison, & Hutson, 2020; Shute, 2021). Psilocybin-assisted therapy (PAP) has arguably generated the most interest and a number of relatively small studies have evaluated its efficacy in treating moderate to severe depression. Some of these studies have included patients with depression with other comorbid conditions, including cancer, AIDS, eating disorders and substance abuse, with promising results (Lea et al., 2020; Ross et al., 2016; Spriggs, Kettner, & Carhart-Harris, 2021). Other studies have compared PAP with more conventional treatments for depression and/or anxiety (Carhart-Harris et al., 2021; Vargas, Meyer, Avanes, Rus, & Olson, 2021). More recently, Goodwin et al. (2022) have compared different doses of psilocybin for TRD: 25 mg, 10 mg or 1 mg, with latter effectively a control.

Studies on PAP to date have generally been small and with limited follow-up. The scale of these studies may be limited by the classification of psilocybin as a schedule 1 drug and the associated costs of handling such substances (Howard, Neill, Schlag, & Lennox, 2021; Rucker, 2015). Additionally, PAP sessions are relatively long and staff-intensive, with the average session lasting five hours with two ‘guides’. However, results of these studies have tended to be positive with efficacy persisting beyond these sessions, which is indicative of the clinical importance of PAP. One clear advantage of psilocybin treatment is that it is generally given only once or twice during an episode of depression, which should have cost implications.

While establishing the efficacy of a particular therapy is essential for guiding clinical choices and policy, it is also important to assess value for money. In England and Wales, the National Institute for Health and Care Excellence (NICE) makes recommendations informed partly by cost-effectiveness using quality-adjusted life years (QALYs). Specifically, NICE utilises incremental cost-effectiveness ratio (ICER) thresholds to inform its guidance on allocation of NHS resources. The ICER is the assumed societal value of one extra QALY. Interventions with an ICER below £20 000 per QALY are often considered to be cost-effective, while those above £30 000 are not. ICERs falling between these two thresholds represent interventions where decision making seems more uncertain. We are unaware of any existing economic evaluations of PAP. The current evaluation is therefore novel and aims to (i) assess the impact of psilocybin-assisted psychotherapy on both service and societal costs (including lost work) and in doing so, (ii) assess its cost-effectiveness. The model builds on the trial of Carhart-Harris et al. (2021) which evaluated the clinical-effectiveness of PAP for long-standing, moderate to severe MDD. We adopt both a healthcare perspective (which is favoured by NICE) and also a societal perspective. The latter includes productivity costs which are likely to be impacted by effective treatments for depression.

## Methods

### Model structure

**Figure 1.**
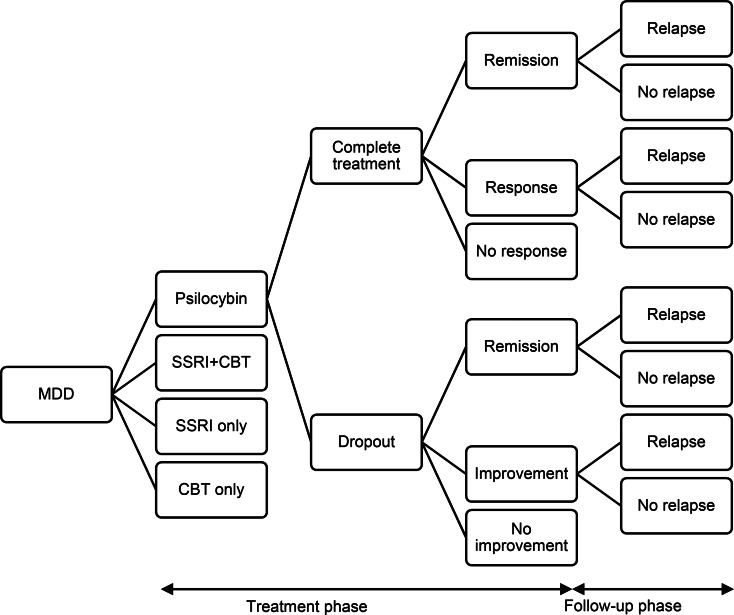
Decision model to compare cost-effectiveness of psilocybin, SSRIs alone, cognitive behavioural therapy alone, and combined SSRIs and cognitive behavioural therapy.

A simple decision tree analytic model was produced in Microsoft Excel to compare different treatment options (Fig. 1 and available from the authors). Such models are used when clinical evidence is available, but cost-effectiveness evidence is lacking. An example of this is the approach used by NICE (National Collaborating Centre for Mental Health, 2010). In the current model, it is assumed that an individual has hard to treat depression and can receive one of four different treatments: PAP, conventional antidepressant treatment alone, cognitive behavioural therapy (CBT) alone, or combined conventional antidepressant treatment and CBT.

The time frame for the model is six months. The initial time period of the model is the acute phase which lasts for six weeks after which there is a twenty-week follow-up phase. It is assumed that a patient will either complete or drop out from treatment. If they complete treatment, it is assumed that they will either have achieved remission from symptoms, responded to treatment but not achieved remission, or not responded. For patients who drop out, it is assumed that there is no treatment response, but spontaneous remission may be achieved, as may improvement without remission or no improvement. During the follow-up phase, patients who achieved remission or sub-remission improvement may relapse or continue in their improved state of health.

### Treatment options

PAP has been implemented in various ways in different studies, with one commonality: psychotherapeutic support is provided to participants before, during, and after treatment. Drawing on this model, the current evaluation initially includes information regarding therapist support from a trial conducted by Carhart-Harris et al. (2021). In this example, participants with chronic medium to severe depression were allocated a pair of therapists (psychologists, psychiatrists, psychotherapists, or psychedelic researchers) to guide them through the therapeutic process. This consisted of one course of treatment: (i) a three-hour preparatory session with both guides, (ii) a one-hour preparatory session with one guide before the second session, (iii) two dosing sessions with 25 mg psilocybin, lasting four to six hours (assumed to be five for the purpose of this evaluation) and accompanied by both guides, (iv) debriefing sessions after each dosing session with both guides present (assumed to be for two hours), and (v) up to three calls after each dosing session with the lead therapist (assumed to last 30 min). It is recognised that this level of support is high and in sensitivity analyses we examine alternative scenarios. The dose of psilocybin was also taken from Carhart-Harris et al. (2021). This was 25 mg given orally on two occasions.

Following the trial, antidepressant therapy was assumed to consist of the selective serotonin reuptake inhibitor escitalopram. The dose was assumed to be 10 mg per day and to last throughout the follow-up period for treatment completers. (The dose did actually increase over time to 20 mg but this has a very small impact on cost.)

Psychological therapy can take many different forms. CBT is widely used in the UK, and therefore was used as the standard treatment in this model. It was also assumed that CBT was delivered across ten sessions by an accredited therapist. Combined antidepressant and psychological therapy was taken to consist of escitalopram and CBT as described above.

### Model parameters

To run the model, we entered values for the probability of events occurring, the costs of the treatment options and subsequent events (e.g. response, remission), and the outcomes from the events in terms of quality of life scores. The parameter values used in the base case model are shown in Table 1. Information on completion of psilocybin and conventional antidepressant treatment, as well as probabilities of remission, response and no response, were taken from the trial conducted by Carhart-Harris et al. (2021). This study compared psilocybin with an SSRI (escitalopram) for people with depression at six weeks’ follow-up. Participants in this study were not resistant to conventional treatment, which was reflected in the 48% treatment response in the SSRI group. From Koeser, Donisi, Goldberg, and McCrone (2015), we obtained a relative risk ratio for dropout, remission, and response for combination therapy for CBT alone compared with conventional medication alone. For combination therapy the relative risks were 0.79 for dropout, 0.86 for response, and 1.27 for remission. For CBT alone the relative risks were 0.62 for dropout, 0.97 for response, and 1.11 for remission. These rates were combined with the probabilities from the Carhart-Harris et al., trial for medication outcomes.

**Table 1.** Model parameters

Parameter	Psilocybin	SSRI	CBT	Combination therapy	Sources
*Probabilities*					
Complete treatment	0.90	0.828	0.89	0.86	Carhart-Harris et al. (2021) Koeser et al. (2015)
Remission by 6 weeks (completers)	0.57	0.28	0.31	0.36	Carhart-Harris et al. (2021) Koeser et al. (2015)
Response by 6 weeks (completers)	0.13	0.20	0.19	0.17	Carhart-Harris et al. (2021) Koeser et al. (2015)
Relapse after remission by 6 months (completers)	0.33	0.55	0.37	0.37	Carhart-Harris et al. (2018) National Collaborating Centre for Mental Health (2010)
Relapse after response by 6 months (completers)	0.33	0.55	0.37	0.37	Goodwin et al. (2018) National Collaborating Centre for Mental Health (2010)
Remission by 3 months (non-completers)	0.10	0.10	0.10	0.10	Assumption
Response by 3 months (non-completers)	0.10	0.10	0.10	0.10	Assumption
Relapse after remission by 6 months (non-completers)	0.50	0.50	0.50	0.50	Assumption
Relapse after response by 6 months (non-completers)	0.50	0.50	0.50	0.50	Assumption
*Costs*					
Psilocybin	£400 to £2000	£0	£0	£0	Assumption
Therapy alongside psilocybin	£3306	£0	£0	£0	Carhart-Harris et al. (2021) Curtis and Burns (2020)
SSRI treatment for 6 months	£0	£22	£0	£0	Joint Formulary Committee (2022)
CBT	£0	£0	£1050	£0	Curtis and Burns (2020)
Combination therapy	£0	£0	£0	£1072	Joint Formulary Committee (2022) Curtis and Burns (2020)
Healthcare during treatment	£1562				Denee et al. (2021)
Healthcare following remission	£783				Denee et al. (2021)
Healthcare following response	£1143				Denee et al. (2021)
Healthcare following no response	£4685				Denee et al. (2021)
Healthcare following relapse	£2342				Denee et al. (2021)
Lost work during treatment	£3123				McCrone et al. (2018)
Lost work following remission	£1566				McCrone et al. (2018)
Lost work following response	£2286				McCrone et al. (2018)
Lost work following relapse	£4685				McCrone et al. (2018)
Lost work following no response	£9369				McCrone et al. (2018)
*Utilities*					
Baseline	0.48	0.48	0.48	0.48	Koeser et al. (2015)
Remission	0.80	0.80	0.80	0.80	Koeser et al. (2015)
Response	0.62	0.62	0.62	0.62	Koeser et al. (2015)
No response	0.48	0.48	0.48	0.48	Koeser et al. (2015)
Relapse	0.48	0.48	0.48	0.48	Koeser et al. (2015)

Relapse rates for psilocybin were obtained from a 6-month study by Carhart-Harris et al. (2018). Relapse rates for antidepressant treatment plus CBT were taken from the NICE guidelines and the rate for CBT alone was assumed to be the same as CBT plus antidepressant treatment (National Collaborating Centre for Mental Health, 2010).

Costs of psilocybin therapy consist of the cost of the drug itself and the cost of therapist support. The cost of the drug will be commercially decided, therefore in these analyses we vary its value from £400 per person upwards in £100 increments to £2000 per person and observe the cost-effectiveness results at each level. The initial cost of inputs from therapists involved multiplying the number of therapist hours (38) from the Carhart-Harris et al., trial by the unit cost of a clinical psychologist. For the latter we assumed a Band 7 clinical psychologist would provide the support and this has a cost per hour of £58 which includes capital and overhead costs (Curtis & Burns, 2020). Thirty minutes of non-direct time was added per treatment hour, resulting in a unit cost of £87. Costs of escitalopram were based on British National Formulary prices (National Institute for Health & Care Excellence, 2022). CBT costs were £1050 for a course (Curtis & Burns, 2020). Healthcare costs (other than the actual therapies) during treatment were not included as they were assumed to be the same for all options. The healthcare costs associated with relapse, remission, and not relapsing were adapted from a recent UK casenote review by Denee et al. (2021). In this analysis, it was assumed, in the absence of better information, that the relapse would occur on average halfway through the 4.5-month post treatment phase. Consequently, the relapse costs from Denee et al., were divided by two, and from this the average costs of treatment following remission or recovery divided by two was subtracted.

Utility values for remission, response, no response, and relapse were taken from Koeser et al. (2015), who used figures originally reported by Kuyken et al. (2008). With probabilities, costs and quality of life scores entered into the model, we calculated expected costs and QALYs for each treatment option. If one option had higher costs and produced more QALYs than an alternative, we calculated an ICER defined as the difference in costs divided by the difference in QALYs.

Costs of lost employment were derived using data from a study of treatment resistant depression (McCrone et al., 2018). This found that costs from lost work were around twice the costs of those for health services, therefore a multiplier of two was used here and applied to healthcare costs.

### Analysis

Although there were four treatment arms in this model, an extended cost-effectiveness analysis was not conducted because the primary focus was on psilocybin-assisted psychotherapy. Therefore, we compare the cost-effectiveness of this with each of the other options separately. We report the incremental cost and QALYs for psilocybin-assisted psychotherapy and where appropriate (i.e. when psilocybin is more expensive and more effective or less expensive and less effective) we report the ICER (incremental cost divided by incremental effectiveness).

As stated earlier, the level of therapist support was assumed to be high. In routine clinical practice it is likely that lower levels of support could be provided or be provided by staff of more junior grades or by non-psychologists. Having said that, while the unit costs of therapists include capital costs they may be underestimated if they do not cover dedicated rooms. We therefore explore the impact of changing the level of support upwards and downwards by 25% and 50% on the cost-effectiveness results. We also conducted similar sensitivity analyses on other parameters. Then, we reported the cost of psilocybin (within the range of £400 to £2000) at which psilocybin is cost-effective (if at all) from the healthcare perspective: (i) remission and response for each therapy, (ii) relapse rates, (iii) cost of psilocybin therapy support, (iv) cost of CBT, (v) healthcare costs following each outcome, (vi) multiplier for lost work costs. The threshold used to determine cost-effectiveness was £20 000 and £30 000, which are the lower- and upper-limits used by NICE (Gandjour, 2020). We also examined the impact on cost-effectiveness from a societal perspective, of reducing the cost of therapist support involved in the therapeutic protocol.

## Results

Over the six-month time period, the expected number of QALYs produced was greatest for psilocybin (0.310), followed by conventional medication plus CBT (0.287), CBT only (0.283), and medication only (0.276). The expected healthcare costs from the initial analyses are shown in Table 2 for different costs of psilocybin. It can be seen that for all costs of the psilocybin itself, from £400 to £2000 per person, the expected healthcare costs are greater for psilocybin therapy (£6255 to £7775) compared with conventional medication only (£3700), CBT only (£4405), and conventional medication plus CBT (£4351).

**Table 2.** Expected healthcare costs

Therapy	Expected cost
Psilocybin (£400 per person)	£6132
Psilocybin (£600 per person)	£6322
Psilocybin (£800 per person)	£6513
Psilocybin (£1000 per person)	£6702
Psilocybin (£1200 per person)	£6892
Psilocybin (£1400 per person)	£7082
Psilocybin (£1600 per person)	£7272
Psilocybin (£1800 per person)	£7462
Psilocybin (£2000 per person)	£7652
SSRI + CBT	£4197
SSRI alone	£3528
CBT alone	£4250

**Figure 2.**
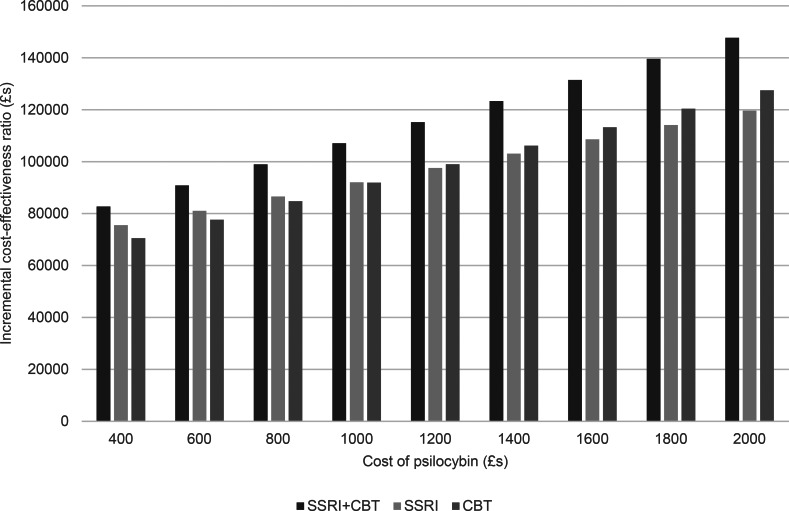
Incremental cost-effectiveness ratio of psilocybin compared to alternative therapies from a healthcare perspective.

**Figure 3.**
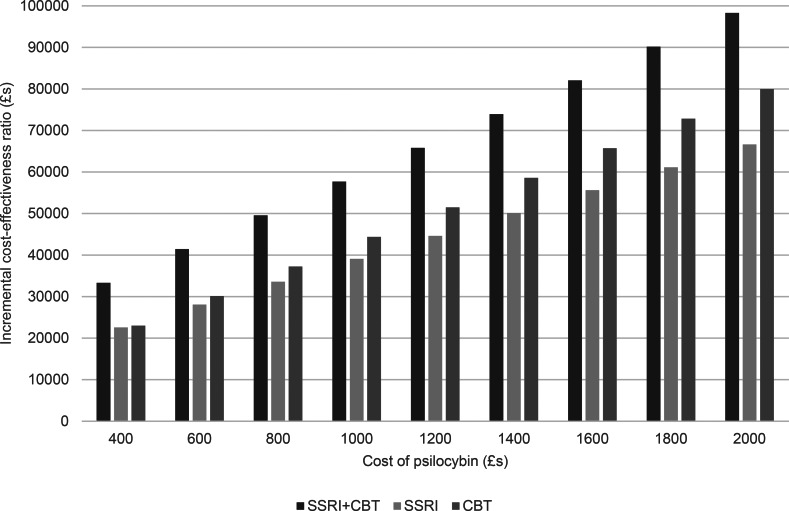
Incremental cost-effectiveness ratio of psilocybin compared to alternative therapies from a societal perspective.

Given the range of psilocybin costs we also have a range of ICERs for each comparison with other therapies as shown by the increase in ICERS shown in Figs 2 and 3 as psilocybin costs rise. Taking a healthcare perspective (Fig. 2), we see that the ICERs are always highest for psilocybin compared to combined medication and CBT. As the cost of psilocybin is increased, the ICERs also increase and initially the ICER for psilocybin compared to medication alone is higher than for psilocybin compared to CBT alone, but this switches when the cost of psilocybin is above £1200 per person. For all psilocybin costs, the ICER is above £30 000 for all comparisons with the high level of therapist support.

When we also consider lost employment costs (Fig. 3), again, the ICER is highest for psilocybin compared to combined medication and CBT. However, at a psilocybin cost of £400 per person, the ICERs are below £30 000 for when compared to medication alone or psychological therapy alone. Additionally, for psilocybin compared to medication alone, the ICER is below £30 000 when the psilocybin cost is £600 per person.

The sensitivity analyses show that, from a healthcare perspective, cost-effectiveness is insensitive to changes in most parameters. However, a key parameter of influence was the cost of therapist support. If this is decreased by 50%, then psilocybin has an ICER below £30 000 compared to combined therapy when the psilocybin cost is £600 per person, to conventional medication alone when the cost is £400 per person, and to CBT alone when the cost is £800 per person. If a threshold of £20 000 is used, then the maximum prices (using the £200 increments) of psilocybin to show cost-effectiveness are £400 against combined therapy and £600 against CBT alone. No price of £400 or above resulted in cost-effectiveness compared to drug treatment alone.

The findings were also influenced by changing the remission rate following psilocybin treatment. If the probability is increased by 50% (i.e. from 0.57 to 0.86) then psilocybin has an ICER below £30 000 when compared to combined therapy at a psilocybin cost of up to £8000 per person, medication alone (£600 per person), and CBT alone (£1000 per person). If a threshold of £20 000 is used, then the maximum prices (using the £200 increments) of psilocybin to show cost-effectiveness in these comparisons are all reduced, and psilocybin is only cost-effective when compared to CBT alone and this only when the price is £400.

When the cost of therapist support is reduced by 50%, the results from a societal perspective show that psilocybin dominates all other options (i.e. it has slower costs and produced more QALYs) up to a cost of £1200 per person. If the cost of therapist support is reduced by just 25%, then psilocybin dominates the other options when the cost per person is £400.

## Discussion

Effective and relatively inexpensive treatments for depression exist, but a significant proportion of people with depression will have an inadequate treatment response and relapse rates can be high. While psilocybin and other psychedelic therapies have been identified as potentially useful treatment options, there are clear challenges in their application. Firstly, psychedelics (including psilocybin) are controlled substances under Schedule 1 of the MDAct1971 and approval for their use in research studies must be specifically obtained. This adds considerable costs at present that will reduce once/if they become approved medicines. In these analyses we have not specifically included the licence costs as we assume in routine practice these will be much reduced at a per person level.

Furthermore, it is recognised that support for patients is required prior to, during, and after the administration of psilocybin, which has large implications for cost-effectiveness. Our modelling study has indicated that if the level of support is at the higher end, then this adversely affects cost-effectiveness. However, in routine practice (i.e. outside the setting of a clinical trial) it is unlikely that each course of PAP would require 38 h of Band 7 therapist time. This model demonstrated the impact on the ICER when therapist support was reduced by 50%, which is easily achieved when a course of PAP is delivered by a single therapist, as opposed to two therapists in the Carhart-Harris et al. (2021) trial. In addition, the model of PAP requires therapists to deliver active therapeutic treatment during preparation and integration sessions only. During the administration of the study medication, therapists are present and supportive, but not delivering active treatment. In routine practice, this support could potentially be provided by Band 5 or Band 6 clinicians, such as those who deliver cognitive behavioural interventions in primary mental health care services.

We have shown that psilocybin can be cost-effective in different scenarios if the therapist support costs are reduced by 25% or by 50%. This is especially the case when taking a societal perspective. Research is currently being carried out on the optimal amount of support and who should provide this (Kettner et al., 2021; Timmermann, Watts, & Dupuis, 2022).. While studies of psilocybin are ongoing, trial-based economic evaluations of alternative levels of therapist support are required. Another option is ‘micro-dosing’ where very small amounts of the drug are taken regularly (perhaps daily). While this presumably may reduce the need for extensive therapist support, there is insufficient evidence as to its effectiveness (Kuypers, 2020).

The drug cost is also influential. When talking about the cost of the drug, we are in reality referring to the price that will be charged were it to be provided commercially. We have explored different scenarios whereby the drug cost (or price) is varied from £400 per person up to £2000 per person. The lower bound of this range is the approximate cost that has been incurred in research studies including that by Carhart-Harris and colleagues (Goldhill, 2018). However, it is highly likely that commercial companies will charge more for the product if it comes to market. What is quite clear is that prices at the high end of this range may make the treatment not cost-effective from a healthcare perspective. It would furthermore be interesting to compare psilocybin with other controlled substances such as ketamine and MDMA.

Assumptions about the remission rate following psilocybin treatment also affect the results. Not surprisingly, if this increases then the cost-effectiveness improves. Based on current trial evidence, achieving such high rates would perhaps be too much to expect. Relapse rate was less influential, but if it is substantially less than assumed here, there may be gains to be had when combined with other changes. It is also worth bearing in mind that PAP is still an emerging area of research, involving a complex intervention in which a number of factors that sit adjacent to the drug and the therapy may influence outcomes (for example, particular forms of music). As research progresses, potential improvements to the delivery of the intervention may have a substantial impact on its efficacy and relapse rates, which would positively influence its cost-effectiveness. Longer follow-up periods will provide more robust evidence.

The perspective taken is crucial. We have shown that there are few circumstances in which psilocybin is likely to be cost-effective from just a healthcare perspective. In England and Wales, NICE prioritises this perspective when making recommendations about new healthcare interventions. Other countries (e.g. the Netherlands) take a broader approach where lost employment costs are included which, when applied here, shows that psilocybin is far more likely to be cost-effective. When the cost of therapist support is reduced, our results show that there are some pricing scenarios where psilocybin dominates other options. However, in the UK there is no recognised threshold at which to assess societal cost-effectiveness as there is with a healthcare perspective. It is clear from a healthcare perspective that PAP is not inexpensive and so better information on long-term outcomes is required. The overall financial impact on the healthcare system depends in large part on who the therapy is best suited for. The Carhart-Harris et al. (2021) trial included those who were not necessarily treatment resistant. If restricted to the latter, that still represents upwards of 12% of people with depression (Andrade et al., 2003) and so the costs could be very high. Taking a societal perspective changes the findings substantially.

### Limitations

It should be considered that the present analyses relied on data from existing clinical studies which were few in number, had short follow-up periods, and included varied groups of patients with depression. The trial on which we based many assumptions had only a six-week follow-up and the participants were potentially less treatment resistant than in other research. The short-term nature of the trial is one reason for using modelling approaches, but it does lead to notable uncertainty in model parameters. Second, many of the cost estimates for treatment outcomes were derived from studies that are now quite old and therapy costs and effectiveness may have changed. Third, the model was a simple decision tree and arguably a more sophisticated approach such as Markov or discrete event simulation modelling could have been used. While these could better have addressed the long-term nature of depression, there are no long-term data on PAP available to run such models. This should be a research priority going forward. Fourth, we only conducted deterministic rather than probabilistic sensitivity analyses. While this is arguably less sophisticated, it did allow us to focus clearly on specific parameter assumptions. Having said this, the deterministic sensitivity analyses were two-way (changing psilocybin prices at the same time as other parameters). Fifth, adverse effects of PAP and the other therapies were not directly costed. However, the included healthcare costs would account for some of these impacts. Sixth, the Carta-Harris et al., trial only compared psilocybin with antidepressant treatment. We used data from other sources for CBT and combination therapy (in order to make broader comparisons) but the lack of direct comparisons in trials may make the results here less certain. Finally, the model relied on a small number of psilocybin studies which produced sometime non-significant results. Use of such data in models is not uncommon and here was the only option. However, it does mean that there is much uncertainty and that evidence base needs improving.

## Conclusions

This study provides preliminary information about the potential cost-effectiveness of psilocybin for treating severe depression. The results indicate positive findings from a societal perspective, which may identify and facilitate more cost-effective approaches to psilocybin therapy. It is essential to better understand who the drug should be prioritised for in terms of treatment resistance and how much therapist support is required.
